# c-MYC expression sensitizes medulloblastoma cells to radio- and chemotherapy and has no impact on response in medulloblastoma patients

**DOI:** 10.1186/1471-2407-11-74

**Published:** 2011-02-16

**Authors:** André O von Bueren, Christoph Oehler, Tarek Shalaby, Katja von Hoff, Martin Pruschy, Burkhardt Seifert, Nicolas U Gerber, Monika Warmuth-Metz, Duncan Stearns, Charles G Eberhart, Rolf D Kortmann, Stefan Rutkowski, Michael A Grotzer

**Affiliations:** 1 Neuro-Oncology Program, University Children's Hospital, Zurich, Switzerland; 2 Department of Pediatric Hematology and Oncology, University Medical Center Hamburg-Eppendorf, Germany; 3 Department of Radiation Oncology, University Hospital Zurich, Zurich, Switzerland; 4 Biostatistics Unit, Institute of Social and Preventive Medicine, University of Zurich, Switzerland; 5 Reference Centre for Neuroradiology, Department of Neuroradiology, University of Wuerzburg, Germany; 6 Department of Pathology, Johns Hopkins University, Baltimore, Maryland, USA; 7 Rainbow Babies and Children's Hospital/Ireland Cancer Center, Cleveland, Ohio, USA; 8 Department of Radiation Oncology, University of Leipzig, Germany

## Abstract

**Background:**

To study whether and how c-MYC expression determines response to radio- and chemotherapy in childhood medulloblastoma (MB).

**Methods:**

We used DAOY and UW228 human MB cells engineered to stably express different levels of c-MYC, and tested whether c-MYC expression has an effect on radio- and chemosensitivity using the colorimetric 3-(4,5-dimethylthiazol-2-yl)-5-(3-carboxymethoxyphenyl)-2-(4-sulfophenyl)-2H-tetrazolium inner salt (MTS) assay, clonogenic survival, apoptosis assays, cell cycle analysis, and western blot assessment. In an effort to validate our results, we analyzed c-MYC mRNA expression in formalin-fixed paraffin-embedded tumor samples from well-documented patients with postoperative residual tumor and compared c-MYC mRNA expression with response to radio- and chemotherapy as examined by neuroradiological imaging.

**Results:**

In DAOY - and to a lesser extent in UW228 - cells expressing high levels of c-MYC, the cytotoxicity of cisplatin, and etoposide was significantly higher when compared with DAOY/UW228 cells expressing low levels of c-MYC. Irradiation- and chemotherapy-induced apoptotic cell death was enhanced in DAOY cells expressing high levels of c-MYC. The response of 62 of 66 residual tumors was evaluable and response to postoperative radio- (14 responders (CR, PR) vs. 5 non-responders (SD, PD)) or chemotherapy (23 CR/PR vs. 20 SD/PD) was assessed. c-MYC mRNA expression was similar in primary MB samples of responders and non-responders (Mann-Whitney U test, p = 0.50, ratio 0.49, 95% CI 0.008-30.0 and p = 0.67, ratio 1.8, 95% CI 0.14-23.5, respectively).

**Conclusions:**

c-MYC sensitizes MB cells to some anti-cancer treatments *in vitro*. As we failed to show evidence for such an effect on postoperative residual tumors when analyzed by imaging, additional investigations in xenografts and larger MB cohorts may help to define the exact function of c-MYC in modulating response to treatment.

## Background

Medulloblastomas (MB) are the most common malignant brain tumors of childhood and constitute 20% of all pediatric brain tumors [1]. Progress in the treatment of MB has been achieved in multiple areas including neurosurgical techniques, refined dosing and delivery of radiation, and optimized chemotherapy [2]. Tumors are currently risk-stratified as standard risk or high risk, depending on clinical factors such as age, extent of resection, and presence of metastases [3].

The oncogene c-MYC is one of the most crucial and frequently deregulated proteins in human cancers [4]. c-MYC is a regulator of S-phase entry, proliferation, and differentiation [5], and has the ability to drive both proliferation and apoptosis [4,6-8]. In situations of cellular stress such as growth factor deprivation, hypoxia, ionizing radiation, or exposure to chemotherapy, c-MYC deregulation may induce apoptosis [9-11]. In childhood MB, high c-MYC mRNA expression, c-MYC gene amplification, and low-level copy changes of c-MYC have been shown to indicate an unfavorable prognosis [12-15], and c-MYC amplification will be used as a molecular stratification factor in the future SIOP-Europe PNET 5 and 6 MB trials.

Several studies investigating whether c-MYC alters the response of cells to radio- and chemotherapy have shown conflicting results [9,11,16-20]. In a recent study we have demonstrated, using small interfering RNA (siRNA) to inhibit c-MYC expression in D341, D425 and DAOY MB cells transiently, that c-MYC down-regulation may reduce sensitivity to radiotherapy, cisplatin, and etoposide treatment [8]. To validate these results and to better understand the effect of c-MYC on MB treatment, we analyzed the response of DAOY and UW228 MB cells engineered to stably express different levels of c-MYC to irradiation and to a panel of chemotherapeutic drugs. We then analyzed c-MYC mRNA expression in formalin-fixed paraffin-embedded tumor samples (FFPE) from well-documented patients with postoperative residual tumor mass treated within the prospective multi-center studies HIT'91 and HIT 2000 and compared c-MYC mRNA expression with response to radio- and chemotherapy, as determined by neuroradiological imaging.

## Methods

### Human MB cell lines

DAOY and UW228, which have not been described to have c-MYC amplifications, were used [21,22]. DAOY wt (wild-type), DAOY V11 (empty vector transfected), DAOY M2 (c-MYC vector transfected), UW228 wt (wild-type), UW228 V1 (empty vector transfected), and UW228 M13 (c-MYC vector transfected) human MB cells have been described previously [23]. The c-MYC cDNA was cloned downstream of the CMV immediate-early promoter [23]. In DAOY M2 and UW228 M13 cells, c-MYC mRNA levels are 15- to 20-fold higher compared with parental or empty vector transfected control cells [23,24]. Protein levels are induced in parallel with mRNA expression and this protein is functional, as shown by 1.5- to 3-fold increases in c-MYC binding activity and transcriptional activity, respectively [23,24]. It has been shown that mRNA expression of DAOY M2 and UW228 M13 cells are comparable to those seen in the upper quartile of primary human MB tumors [23]. We verified c-MYC expression of DAOY and UW228 cell lines by real-time quantitative RT-PCR and western blot analysis repeatedly. All DAOY MB cells were cultured in Richter's zinc option medium/10% fetal bovine serum. All UW228 MB cells were grown in DMEM/F12, 10% fetal bovine serum (G418 was added to the medium for DAOY V11, DAOY M2, UW228 V1, and UW228 M13 to a concentration of 500 μg/ml). All cells were incubated at 37°C in a humidified atmosphere with 5% CO_2_.

### Western blot analysis

The protein expression of caspase-8, caspase-9, and β-actin was assessed by Western blot analysis. In brief, cell lysates were obtained from DAOY wt, DAOY V11, and DAOY M2 MB cells following different treatments (irradiation (IR), cisplatin, and etoposide). Whole-cell pellets were lysed, 40 μg total protein was separated by 10% SDS polyacrylamide gels, and the gels were subjected to immunoblotting as described previously [8,24]. Nonspecific binding sites were blocked by 3 h incubation in TBST (10 mM Tris pH 8.0, 150 mM NaCl, 0.05% Tween 20) supplemented with 5% non-fat milk powder. Membranes were incubated overnight at 4 °C with a 1:200 dilution of rabbit polyclonal anti-caspase-8 antibody (Santa Cruz Biotechnology; Heidelberg, Germany) and with a 1:1000 dilution of rabbit polyclonal anti-caspase-9 antibody (Cell Signaling; Allschwil, Switzerland). Membranes were then washed three times at room temperature in TBST for 30 min each time, and bound Ig was detected using anti-isotype monoclonal secondary antibody coupled to horseradish peroxidase (Santa Cruz Biotechnology; Heidelberg, Germany). The signal was visualized by enhanced chemiluminescence ECL (Amersham Biosciences; Dübendorf, Switzerland) and autoradiography. Immunoblotting with a 1:5000 dilution of a monoclonal primary β-actin antibody (Sigma; Basel, Switzerland) was performed to verify equivalent amounts of loaded protein.

### Chemo- and radio-sensitivity assays

Appropriate numbers of exponentially growing MB cells were seeded in 96-well plates. Cells were treated for 72 h with various concentrations of cisplatin, carboplatin, doxorubicin, etoposide, methotrexate, or vincristine, or were irradiated with 2, 5, or 10 Gy; using a Pantak Therapax 300 kV X-ray unit at 0.7 Gy/min. Dosimetry was controlled with a Vigilant dosimeter. Cell viability of human MB cells was quantified using the colorimetric 3-(4,5-dimethylthiazol-2-yl)-5-(3-carboxymethoxyphenyl)-2-(4-sulfophenyl)-2H-tetrazolium inner salt (MTS) assay 72 h after IR or after adding the chemotherapeutic drugs [8,24]. Each experiment was performed in sixplicate. All assays were repeated as independent experiments at least twice.

### Clonogenic survival assay

To assess clonogenic survival, the number of single seeded cells was adjusted to obtain around 100 colonies per cell culture dish with a given treatment, as described previously [8]. After exposure to the different treatments, cells were maintained at 37 °C in a humidified atmosphere containing 5% CO_2 _and allowed to grow for 9-10 days before fixation in methanol/acetic acid (75%:25%) and staining with Giemsa. Colonies with more than 50 cells were counted. Each experiment was performed in triplicate. All clonogenic assays were repeated as independent experiments at least twice.

### Cell cycle analysis

After treating the human MB cells with IR or cytotoxic drugs, both floating and adherent cells were collected. After washing twice in phosphate-buffered saline (PBS), the cells were stained with a solution containing 50 μg/ml propidium iodide (Becton-Dickinson; Allschwil, Switzerland) and 100 U/ml RNase A (Qiagen; Hombrechtikon, Switzerland) in PBS for 30 min at room temperature. The percentage of cells in the different phases of the cell cycle was assessed by evaluating DNA content as described previously [8,24].

### Apoptosis assay

A photometric enzyme-immunoassay (Cell Death Detection ELISA; Roche Diagnostics, Basel, Switzerland) was used for the quantitative determination of cytoplasmic histone-associated DNA fragments, as described elsewhere [8,24]. Each experiment was done in triplicate.

### MB patients and therapy

Children with MB were treated within the two consecutive prospective multicenter trials HIT'91[25] and HIT 2000 (ClinicalTrials.gov**/**NCT00303810). In total, 280 patients with histologically proven MB, diagnosed between 1991 to 1997, aged 3 to 18 years, were randomized to treatment by postoperative chemotherapy followed by radiotherapy versus postoperative radiotherapy followed by maintenance chemotherapy (HIT'91) [25]. FFPE tumor samples to assess c-MYC mRNA expression were available from 31 of 81 MB patients with postoperative residual tumor.

Children with non-metastatic MB diagnosed since January 2001, aged 4 to 21 years, were treated by postoperative radiotherapy followed by maintenance chemotherapy [25-27] (HIT 2000 AB4-SIOP PNET 4), and patients with metastatic MB by postoperative chemotherapy, two cycles of HIT-SKK [28], followed by radiotherapy and maintenance chemotherapy [25-27] (MET-HIT 2000 AB4), as described previously [29]. FFPE tumor samples to assess c-MYC mRNA expression were available from 37 of 105 MB patients with postoperative residual tumor (out of 275 MB patients, at the time of analysis). All tumors - analyzed for c-MYC mRNA expression (n = 68) - were resected from patients who had not received any treatment before. Postoperative residual tumor was evaluated by MRI or CT within 72 hours after surgery.

All institutions participating in the studies had received approval from their institutional review boards and informed consent was obtained from legal representatives of patients. Staging within the studies was performed according to Chang criteria [30]. Central histopathological, neuroradiological, and cerebrospinal fluid (CSF) analysis was recommended for all patients. Patients enrolled in the studies were evaluated by neuroimaging studies of the brain - contrast-enhanced CT, gadolinium-enhanced MRI, or both - to determine the response to radiation therapy and chemotherapy using radiographic criteria as described previously [25,28]. Complete response (CR) was defined as complete resolution of tumor as assessed by neuroimaging studies, partial response (PR) was defined as a decrease in tumor size by ≥ 50%, stable disease (SD) as a decrease of less than 50% or an increase of less than 25%, and progressive disease (PD) as an increase of ≥ 25%. Responders were defined as children with CR/PR, and non-responders as patients with SD/PD.

### RNA isolation and quantitative reverse transcription-PCR for c-MYC

Isolation of total RNA from FFPE tumor tissue and real-time quantitative RT-PCR for analyses of c-MYC have been described previously [13].

### Statistical analysis

All data are expressed as mean ± standard deviation. Differences between groups were examined for statistical significance using the unpaired Student's t-test. *P *< 0.05 was in general considered to be significant. GraphPad Prism 4 (GraphPad Software, San Diego, California) software was used to calculate IC_50 _values and their 95% confidence intervals and to statistically compare fitted midpoints (log IC_50_) of the two curves [8]. The Mann-Whitney test was used to compare c-MYC mRNA expression of tumors responding (CR/PR) with tumors not responding (SD/PD) to first treatment modality (radiotherapy or chemotherapy). SPSS Software 17.0 was used for univariate and multivariate Cox regression analysis to investigate whether c-MYC mRNA expression is associated with duration to best response. c-MYC mRNA expression was scored as a continuous variable (log10 transformed to obtain an approximately normally distributed variable, 0.01 added if c-MYC mRNA level was not detectable). The central tendencies of c-MYC mRNA levels for responders and non-responders were calculated on the basis of log-transformed data, i.e. ratios of geometric means with 95% confidence intervals (CI) are reported.

## Results

### Susceptibility of DAOY and UW228 cells expressing different levels of c-MYC to IR, etoposide, cisplatin, carboplatin, doxorubicin, methotrexate, and vincristine

**Figure 1:**
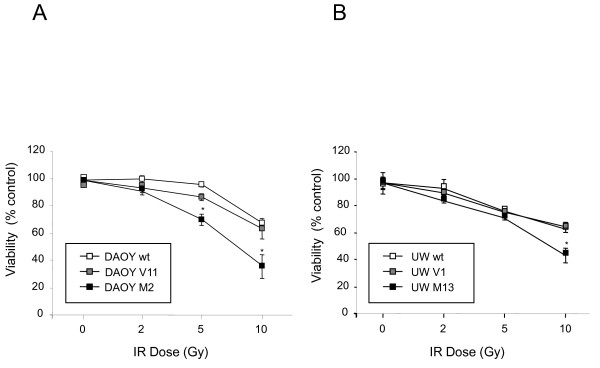
**Effect of c-MYC expression on radio-sensitivity of DAOY and UW228 MB cells**. DAOY (c-MYC over-expressing) cells **(A) **and less pronounced UW228 (c-MYC over-expressing) cells **(B) **are more susceptible to IR as determined by MTS assay compared with control cells (DAOY V11; UW228 V1). Values represent the mean percentage of viability (representative from two independent experiments) compared with non-irradiated cells ± standard deviation (n = 6). Student's t-test compared DAOY M2 and UW228 M13/irradiated cells vs. DAOY V11 and UW228 V1/irradiated cells (***: P < 0.001, **: P < 0.01,*: P < 0.05).

**Figure 2:**
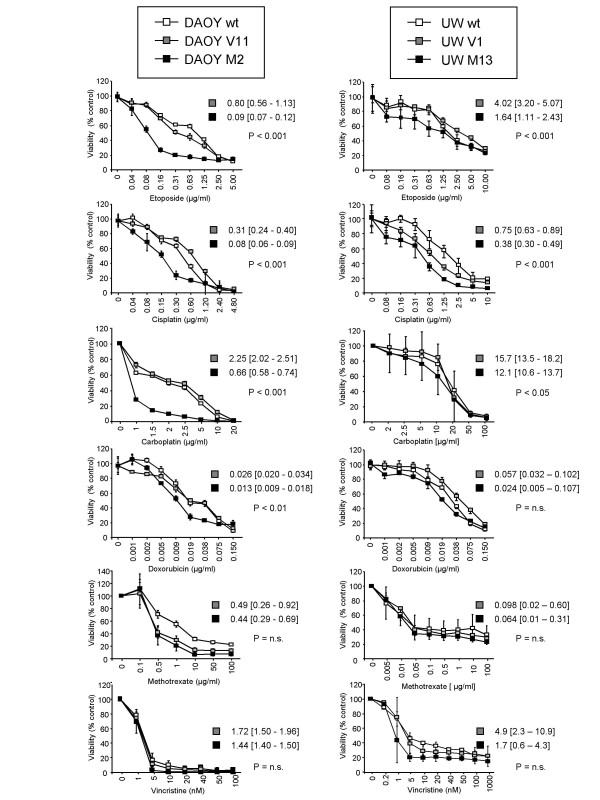
**Effect of c-MYC expression on chemo-sensitivity of DAOY and UW228 MB cells**. DAOY (c-MYC over-expressing) and to a lesser extent UW228 (c-MYC over-expressing) cells are more susceptible to etoposide, cisplatin, carboplatin, and doxorubicin, and are equally sensitive to methotrexate and vincristine, as determined by MTS assay compared with control cells (DAOY wt, V11; UW228 wt, V1). Values represent the mean percentage of viability (representative from two independent experiments) compared with non-treated cells ± standard deviation (n = 6). The IC_50 _values and their 95% confidence intervals were calculated from the regression curve and are indicated for each data set for c-MYC- and empty vector-transfected DAOY and UW228 cells. Differences between the two curves are represented by P-values (P < 0.001, P < 0.01, P < 0.05, n.s. not significant).

To investigate whether the sensitivity of MB cells to radio- and chemotherapy depend on the c-MYC expression level, we assessed cell viability of DAOY and UW228 human MB cells expressing different levels of c-MYC 72 h following irradiation with 0, 2, 5, or 10 Gy (Figure 1), or 72 h after treatment with different concentrations of etoposide, cisplatin, carboplatin, doxorubicin, methotrexate, or vincristine (Figure 2). IR (10 Gy) decreased viability to 68.0 ± 1.9% in DAOY wt, to 63.2 ± 7.7% in DAOY V11, and to 34.7 ± 9.2% in DAOY M2 (c-MYC over-expressing) cells; IR (10 Gy) reduced viability to 67.5 ± 2.1% in UW228 wt, to 69.1 ± 3.5% in UW228 V1, and to 49.5 ± 5.2% in UW228 M13 (c-MYC over-expressing) cells.

**Figure 3:**
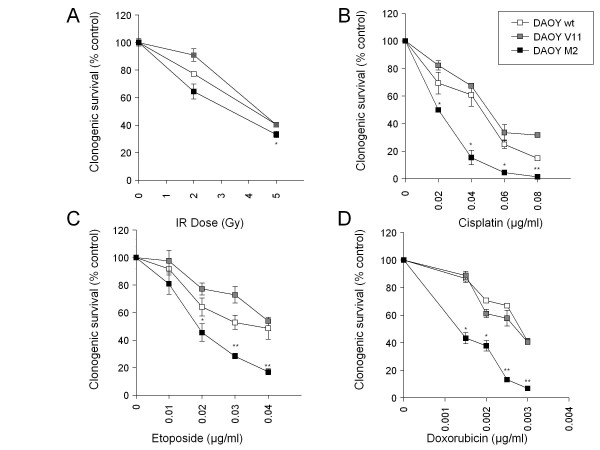
**Clonogenic survival of DAOY MB cells after IR, cisplatin, etoposide, and doxorubicin**. The clonogenic survival of DAOY (c-MYC over-expressing) cells following IR **(A)**, cisplatin **(B)**, etoposide **(C)**, and doxorubicin **(D) **is more compromised as assessed by clonogenic survival assay compared with control cells (DAOY wt, V11). Values represent the mean percentage of clonogenic survival (representative from two independent experiments) compared with non-irradiated or solvent-only treated cells ± standard deviation (n = 3). Student's t-test compared DAOY M2/treated cells vs. DAOY V11/treated cells (***: P < 0.001, **: P < 0.01,*: P < 0.05).

DAOY M2 and UW228 M13 cells were significantly more susceptible to etoposide and cisplatin (p < 0.001), DAOY M2 cells also to carboplatin (p < 0.001), and DAOY M2 and UW228 M13 cells tend to be more responsive (not significant) to doxorubicin and vincristine when compared to corresponding empty vector transfected cells (DAOY V11 and UW228 V1) (Figure 2). DAOY and UW228 cells expressing different levels of c-MYC were equally sensitive to methotrexate. In particular, DAOY M2 cells appear to be more sensitive to IR, platins, and topoisomerase II inhibitors as determined by the MTS assay when compared with control cells. Therefore, differences in cellular sensitivity were further investigated using clonogenic survival assays to determine the susceptibility of DAOY (wt, V11, and M2) cells to IR (Figure 3A), cisplatin (Figure 3B), etoposide (Figure 3C), and doxorubicin (Figure 3D). The clonogenic survival of DAOY M2 cells was more reduced after IR, cisplatin, etoposide, and doxorubicin when compared with control cells. Taken together, our results show that c-MYC levels do affect cellular sensitivity to IR and selected chemotherapeutics.

### Irradiation-, cisplatin-, etoposide-, and doxorubicin-induced cell cycle alteration in DAOY MB cells expressing different levels of c-MYC

**Figure 4:**
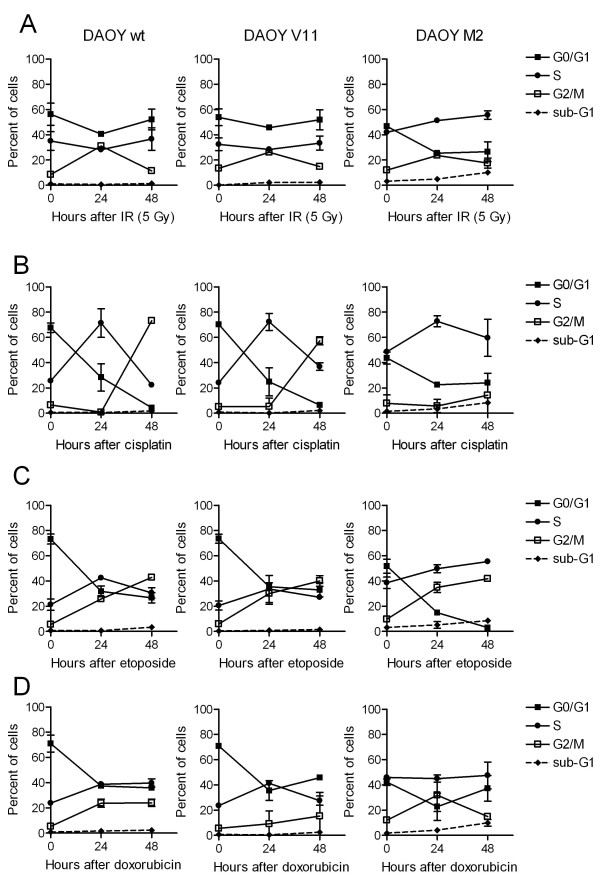
**IR-, cisplatin-, etoposide-, and doxorubicin-mediated effect on cell cycle progression in DAOY MB cells**. Cell cycle analysis following IR **(A)**, exposure to cisplatin **(B)**, etoposide **(C)**, and doxorubicin **(D)**. DAOY MB cells were irradiated with 5 Gy or treated with cisplatin (0.15 μg/ml), etoposide (0.19 μg/ml), or doxorubicin (0.02 μg/ml), and subjected to cell cycle determination by propidium iodide staining and FACS analysis at the time points indicated. The percentages of cells in G1, S, G2/M, and sub-G1 phases of the cell division cycle are indicated ± standard deviation (n = 2).

To study whether treatment-induced effects on cell cycle progression and induction of apoptosis differ in DAOY cells, we exposed DAOY (wt, V11, and M2) MB cells to IR. Subsequently, the cells were cultured for up to 48 h and subjected to determination of cell cycle profile by fluorescence activated cell sorter (FACS) analyses. As shown in Figure 4A, all DAOY cells arrested in the G2/M phase of the cell cycle about 24 h after irradiation (5 Gy). The G2/M arrest did not appear to be irreversible as cells re-entered the cell cycle, as suggested by the reappearance of a G1 peak within 48 h after IR. We observed IR (5 Gy) mediated sub-G1 accumulation in DAOY M2 but not in DAOY wt and DAOY V11 cells (Figure 4A). To investigate the cell cycle response to treatment with cisplatin, etoposide, and doxorubicin, DAOY (wt, V11, and M2) MB cells were exposed to the different chemotherapeutic agents under conditions where differences in short-term toxicity were observable (Figure 2). As shown in Figure 4B, DAOY cells initially arrested in S-phase after 24 h and then in the G2/M phase of the cell cycle about 48 h after cisplatin treatment. On the other hand etoposide and doxorubicin induced a G2/M-arrest in DAOY (wt, V11, and M2) MB cells after 24-48 h of treatment (Figure 4C,D). Strikingly, sub-G1 accumulation was more prominent in DAOY M2 cells after exposure to the different treatments (Figure 4A-D). In summary, IR induced a reversible G2/M-arrest in all DAOY cells followed by re-entry into the G1 phase. The cytotoxic drugs tested in this study induced a transient arrest in the S-phase (cisplatin) and in the G2/M-phase (cisplatin, etoposide, and doxorubicin) in all DAOY cells. Importantly, sub-G1 accumulation, which suggests apoptosis, was induced after IR and selected cytotoxic drugs mainly in DAOY M2 cells.

### Irradiation-, cisplatin-, etoposide-, and doxorubicin-induced apoptotic cell death is increased in DAOY c-MYC over-expressing cells

**Figure 5:**
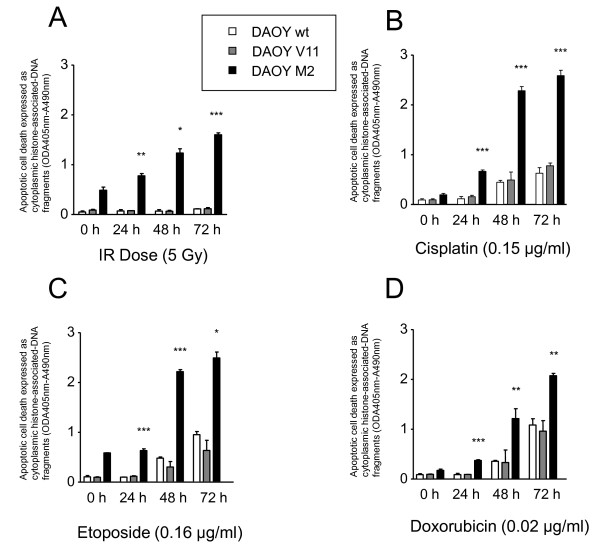
**Effect of c-MYC expression on the apoptotic activity of DAOY MB cells after exposure to DNA-damaging agents**. IR **(A)**, cisplatin **(B)**, etoposide **(C)**, and doxorubicin **(D) **enhance the induction of apoptotic cell death in c-MYC over-expressing DAOY cells, as determined by Cell Death ELISA assay at the different time points indicated. Values represent the mean absorbance of cytoplasmic histone-associated-DNA fragments ± standard deviation (n = 3). Student's t-test compared DAOY M2/treated cells vs. DAOY V11/treated cells (***: P < 0.001, **: P < 0.01,*: P < 0.05).

To test whether treatment-induced apoptosis differs in DAOY (wt, V11, and M2) MB cells, apoptotic cell death was assessed using Cell Death ELISA at 0, 24, 48, and 72 h after IR (5 Gy), cisplatin, etoposide, and doxorubicin treatment under conditions where differences in short-term cytotoxicity was detectable (Figure 2). DAOY M2 cells are characterized by higher basal apoptotic activity compared with DAOY wt and DAOY V11 (Figure 5). After treatment with IR (5 Gy) apoptotic cell death was induced in DAOY M2 cells in a time-dependent manner, whereas IR (5 Gy) did not induce apoptosis in DAOY wt and V11 cells (Figure 5A). Likewise, a high c-MYC level enhanced susceptibility to cisplatin- (Figure 5B), etoposide- (Figure 5C), and doxorubicin- (Figure 5D) induced apoptotic cell death in a time-dependent manner. Chemotherapy induced apoptotic cell death to a notably lesser extent in the parental and empty vector-transfected DAOY cells when compared with DAOY M2 cells. In conclusion, these results confirm that c-MYC over-expression increases basal apoptotic activity and the susceptibility to undergo apoptosis after treatment with various cytotoxic agents.

### c-MYC acts in synergy with irradiation, cisplatin, and etoposide to trigger caspase-9 cleavage in DAOY c-MYC over-expressing cells

**Figure 6:**
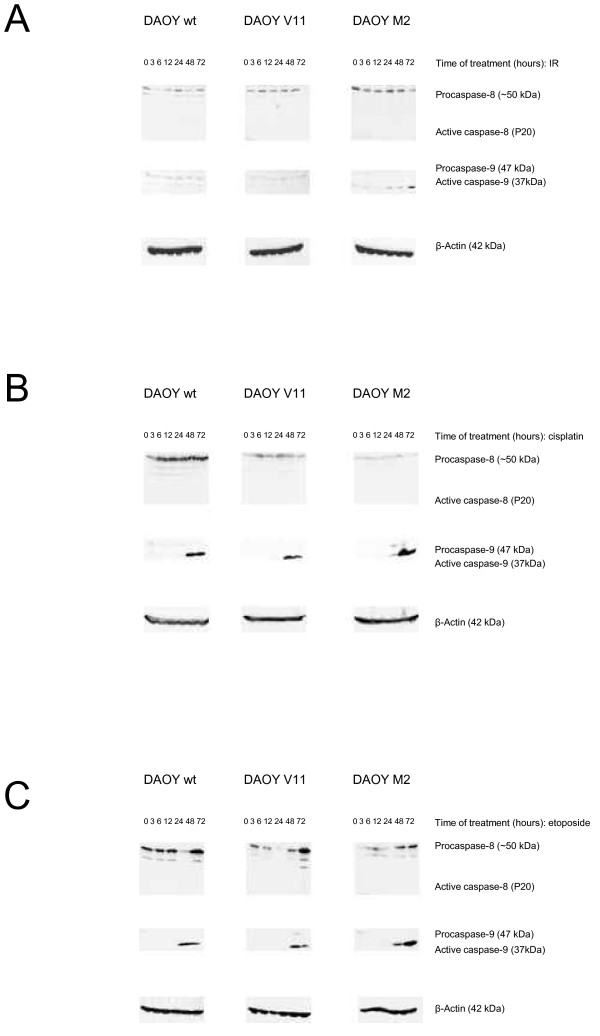
**c-MYC expression modulates caspase-9 activation after IR, cispatin, and etoposide treatment of DAOY MB cells**. IR **(A) **triggers caspase-9 cleavage exclusively in c-MYC over-expressing DAOY cells. Caspase-9 cleavage is more pronounced in DAOY M2 cells following treatment with cisplatin **(B)**, or etoposide **(C)**. Cells were irradiated with 5 Gy, treated with cisplatin (0.15 μg/ml) or with etoposide (0.19 μg/ml). Lysates were analyzed at the indicated time points by Western blotting for caspase 8 and 9, and β-actin. Representative blots (two independent experiments) are shown.

We next investigated whether IR, cisplatin, and etoposide induce caspase-dependent apoptotic cell death. We assessed the processing of pro-caspase 8 and 9 after IR, cisplatin, and etoposide treatment. As shown in Figure 6A, IR (5 Gy) of DAOY (wt, V11, and M2) cells resulted in the proteolytic cleavage of pro-caspase 9 only in DAOY M2 cells. No proteolytic cleavage of pro-caspase 8 was detected in DAOY (wt, V11, and M2) cells. We also assessed the effect of cisplatin and etoposide on the activation of pro-caspase 8 and pro-caspase 9 in DAOY (wt, V11, and M2) cells, and found that processing of pro-caspase 9 was more pronounced in DAOY M2 cells (Figure 6B,C). Pro-caspase 8 cleavage was not detected in any DAOY (wt, V11, and M2) cells after treatment with cisplatin or etoposide.

### Response of postoperative residual MB tumors to radio- and chemotherapy and outcome according to c-MYC mRNA expression

Our *in vitro *experiments provide evidence that c-MYC sensitizes MB cells to some anti-cancer treatments. In order to investigate whether our observations might be of clinical relevance, we analyzed whether the response of postoperative residual tumor to radio- and chemotherapy depends on the c-MYC mRNA expression in MB patients. Adequate material for assessment of c-MYC mRNA expression was available for tumors of 68 MB patients. In our current analysis we included 31 MB patients (> 3 years of age) from our previous study [13] with incomplete surgical resection, and an additional 37 MB patients (> 4 years of age) with incompletely resected tumors treated within the prospective multi-center study HIT 2000. In 66 of these patients the quality of isolated RNA, based on quality assurance described previously [13], was sufficient for determining c-MYC mRNA expression. The amount of c-MYC mRNA expression was related to human cerebellum [13] and a cut-off value of 1 was chosen to define patient groups with high or low expression of c-MYC, as described elsewhere [13]. Differences in overall survival (OS) of 66 patients (median follow-up: 3.1 years, median age: 7.4 years), as determined by univariable analysis, between patients with high (n = 37) and low (n = 29) c-MYC mRNA expression (3-year OS 46 ± 10% versus 76 ± 10%, *p *= 0.10), were comparable to our previous study [13].

**Figure 7:**
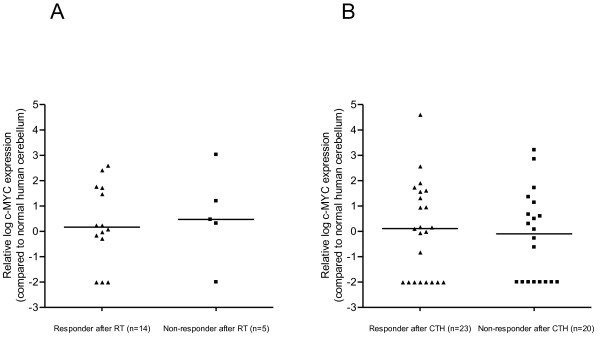
**c-MYC mRNA expression of responsive versus non-responsive MB tumors to postoperative radiotherapy (RT) or chemotherapy (CTH)**. c-MYC mRNA expression of MB tumors responding to postoperative RT **(A) **or postoperative CTH **(B) **were not significantly different to MB tumors failing to respond during the first postoperative treatment modality as determined by Mann-Whitney U test.

Neuroradiological evaluation and c-MYC mRNA expression analysis was of sufficient quality to determine response rate on the residual tumor, as described elsewhere [25], according to c-MYC mRNA expression following treatment by postoperative radiotherapy (19/21 patients) and by postoperative chemotherapy (43/45 patients) (Figure 7) as reported previously [31]. c-MYC mRNA expression in tumors from 14 responders (CR/PR) after postoperative radiotherapy was not significantly different compared with c-MYC mRNA expression in tumors from 5 non-responders (SD/PD) as determined by Mann-Whitney U test (*p *= 0.50, ratio 0.49, 95% CI 0.008-30.0). No significant difference in c-MYC expression was found in tumors from 23 responsive patients and c-MYC expression in tumors from 20 non-responsive patients after postoperative chemotherapy (Mann-Whitney U test, *p *= 0.67, ratio 1.8, 95% CI 0.14-23.5). The effect of c-MYC mRNA level on time to best objective response was assessed using univariate and multivariate Cox regression analysis. Among the 66 patients with postoperative residual tumors who had c-MYC mRNA expression analysis, 65 were evaluable for best response: 3 (5%) PD, 3 (5%) SD, 8 (12%) PR, and 51 (78%) CR. Best response was not evaluable in one case because the quality of postoperative MRI was insufficient. Median time to best objective response was 0.38 years (0.07-1.34). Increasing tumor tissue c-MYC mRNA levels were not significantly associated with an increasing probability of objective response (p = 0.62; hazard ratio 1.04; 95% CI 0.90-1.20). In addition to c-MYC mRNA level, age, gender, histology, and the presence or absence of metastasis were included in a multivariate model. No covariates were significantly associated with time to response (data not shown).

## Discussion

The impact of c-MYC (c-MYC gene amplification and c-MYC mRNA expression) on the prognosis of MB has been thoroughly investigated [12-14], but the effect of c-MYC on MB standard treatment remains largely unknown. The present study is the first to investigate the relationship between c-MYC expression and treatment response in MB patients and analyzes the cell-death promoting effect of clinically relevant chemotherapeutic drugs and IR on two MB cell lines engineered to stably express different levels of c-MYC.

DAOY M2 and UW228 M13 human MB cells, stably engineered to over-express c-MYC, proved more sensitive to some cytotoxic drugs and IR when compared with wild-type and empty vector transfected control cells. A series of preclinical studies suggested that c-MYC may sensitize a variety of cells to different treatments [9,11,16]. Over-expression of c-MYC is associated with enhanced susceptibility to etoposide-, doxorubicin-, and cisplatin-induced apoptosis [11], whereas loss of c-MYC expression reduced etoposide- and doxorubicin-mediated apoptotic cell death [17]. However, there are also reports showing that a decrease of c-MYC level may sensitize human melanoma cells to cisplatin [19,20]. Our current findings, as well as our previous study [8] using siRNA to down-regulate c-MYC expression transiently in MB cell lines (DAOY, D341, and D425), provide evidence that c-MYC may increase the cytotoxicity of IR and selected chemotherapeutic drugs to MB cells [8].

DAOY cells, engineered to express different levels of c-MYC, showed differing sensitivity to IR, platins, etoposide, and were used to further investigate how c-MYC may modulate the cellular response to IR and some chemotherapeutic drugs. Our study shows that differences in cell cycle alterations appear unlikely to be responsible for the different radio- and chemo-sensitivity of DAOY cell lines. IR and DNA-damaging chemotherapies resulted in a comparable pattern of cell-cycle check-point activation in the three DAOY cell lines (wt, V11, and M2). In all DAOY cells, IR resulted in a transient G2/M-arrest, allowing DNA double-strand break repair [32]. A similar G2/M-arrest (24-48h) was observed after treatment with the topoisomerase II poisons (etoposide and doxorubicin) in all three DAOY cell lines. Treatment with cisplatin initially resulted in a transient S-phase arrest (24 h) and subsequently in a G2/M arrest (48 h) in all these DAOY cell lines. This observation is in agreement with studies demonstrating that cisplatin induces a transient S-phase arrest, followed by inhibition of the Cdc2-cyclin A or B kinase to induce a durable G2/M arrest [33]. A possible explanation for the different sensitivity to DNA-damaging agents of DAOY cells expressing various c-MYC levels is that it might be related to differing susceptibility to the induction of apoptosis. Interestingly, we consistently found that IR as well as cytotoxic compounds induced an increase in the proportion of cells in sub-G1, mainly in DAOY M2 cells. To investigate whether DAOY M2 cells are more prone to DNA damage-induced apoptotic cell death, we assessed the apoptotic response of DAOY (wt, V11, and M2) cells following IR, cisplatin, etoposide, and doxorubicin more quantitatively, as described elsewhere [8,24]. We found that IR-induced apoptosis was only evident in DAOY M2 cells whereas chemotherapy-induced apoptosis was more prominent in DAOY M2 cells compared with DAOY wt and DAOY V11 cells. These results are supported by Dee et al., who demonstrated that IR (4 Gy) failed to induce apoptotic cell death in DAOY (wild-type) cells [34]. Furthermore, DAOY cells engineered to over-express c-MYC render p53 mutant DAOY cells [35] susceptible to the induction of apoptosis. High c-MYC expressing DAOY cells were also more susceptible to chemotherapy-induced apoptosis. These findings are supported by studies showing that c-MYC may augment IR- and chemotherapy-induced apoptosis [9,11,16,17]. Conversely, c-MYC down-regulation resulted in an increase of cisplatin-induced apoptotic cell death in human melanoma cells [19,20], suggesting that the decisive factors influencing a cell to undergo apoptotic cell death and how c-MYC regulates this apoptotic response depend on the cell type [36]. In p53 mutant DAOY cell lines, c-MYC over-expression appears to amplify the intrinsic mitochondrial, but not the extrinsic, apoptotic pathway [36], as suggested by the absence of pro-caspase 8 cleavage in DAOY (wt, V11, and M2) cells and more pronounced activation of pro-caspase 9 in DAOY M2 compared with DAOY wt and V11 cells following IR, cisplatin, or etoposide.

In MB patients, it has been shown that high c-MYC mRNA expression is unfavorably associated with prognosis [12,13]. We analyzed whether c-MYC expression, determined in the primary MB tumors, has an effect - as determined by neuroradiological evaluation - on the response of residual tumor to postoperative IR and postoperative chemotherapy. We found that the c-MYC mRNA expression in tumors from 14 children (74%) who responded to postoperative IR were similar and not significantly different from c-MYC expression in tumors from 5 patients (26%) who did not respond. The response rate of residual tumors to postoperative IR was comparable to response rates observed in the HIT'91 trial which reported an objective postoperative IR response rate for residual tumors of 73.6% [25]. c-MYC mRNA expression in tumors from 23 responders (53%) to postoperative chemotherapy was not significantly different from c-MYC mRNA expression in tumors from 20 non-responders (47%) [25,28]. It is well known that MB is uniquely sensitive to chemotherapy and radiation [2]. We therefore also investigated whether c-MYC determines time to response but failed to detect any influence using univariate or multivariate Cox regression analysis.

Some care should be taken in the interpretation of the results, since our study has several limitations. The relationship between the response to postoperative IR and c-MYC mRNA expression was studied in only a small number of patients (14 responders versus 5 non-responders). The relationship between chemo-response and c-MYC mRNA expression was studied in 43 patients (23 responders versus 20 non-responders) treated according to two different regimens [25,29]. These limitations, including the use of polychemotherapy, restrict the evaluation of the effect of c-MYC mRNA expression on responses to individual chemotherapy and a potential relationship between an individual drug and c-MYC mRNA expression cannot be excluded. Iba et al. found that responders to the treatment with platinum compounds among patients with epithelial ovarian cancer had higher c-MYC levels compared with non-responders, resulting in even higher survival rates [31].

## Conclusions

Taken together, c-MYC moderately enhances the anti-tumor effect of several DNA-damaging agents through sensitization to apoptosis in c-MYC over-expressing DAOY MB cells. These results provide important insights into mechanisms determining the sensitivity of MB cells to DNA-damaging agents. Although high c-MYC expression moderately sensitizes MB cells to DNA-damaging agents, we were unable to find evidence for this effect by analyzing neuroradiological imaging of postoperative residual tumors. Our results provide evidence that the unfavorable prognosis of MB patients with high c-MYC expressing tumors may not be explicable by their poor response to treatment, although additional validations in xenografts and a larger MB cohort is necessary to clarify the exact role of c-MYC in modulating treatment response. So far, poor outcome of MB patients with high c-MYC expressing tumors appears to be explainable by their aggressive clinical nature, the tendency of high c-MYC expressing MB cells to rapid growth [8,23,37], the fact that high c-MYC mRNA expressing MB tumors are often associated with tumor anaplasia [23] and relapse more frequently [12,13]. Our study shows that MB cells expressing high c-MYC levels are sensitive to IR and some chemotherapeutic drugs and provides evidence that a more aggressive treatment approach might be considered in the future to improve the outcome in those patients.

## Competing interests

The authors declare that they have no competing interests.

## Authors' contributions

Study concepts/design: AOVB, CO, TS, MAG. Data aquisition: AOVB, CO, MWM, KVH, RDK, SR. Data analysis und interpretation: AOVB, CO, TS, KVH, MP, BS, NUG, MWM, DS, CGE, RDK, SR, MAG. Statistical analysis: AOVB, BS. Manuscript writing: AOVB. Manuscript editing: CO, MAG. Manuscript review: All authors. All authors read and approved the final manuscript.

## Pre-publication history

The pre-publication history for this paper can be accessed here:

http://www.biomedcentral.com/1471-2407/11/74/prepub
